# Supplementary material for: Evaluation Using Polar Coordinate of the Representation of Movement in the Drawings of Children Aged 5 to 8 Years
Source: Int J Environ Res Public Health. 2022 Mar 1;19(5):2844. doi: 10.3390/ijerph19052844 (PMC8910050; doi:10.3390/ijerph19052844)

Supplementary Material

Evaluation using polar coordinate of the representation of movement in the drawing of children aged 5 to 8 years

Table S1. Indicators of graphic representation of movement with very significant relationships (P <0.001) between focal and conditione behavior.

5 AGE								
Character Rabbit	Macrocategory	Category	Focal behavior	Conditioned behavior	Significant vectors			
					Quadrant	Length	Angle	Significance
	Estatic	Vertical-front	1_evfcf	3_evfbcr	I	2,87	85,62	**
				2_evfbc	III	2,76	268,34	**
			2_evfbc	1_ehpcfpj	I	2,76	1,66	**
				2_ehpcfpf	I	2,76	1,66	**
				1_evfcf	III	2,76	181,66	**
				4_evfpj	III	2,76	181,66	**
			3_evfbcr	1_evfcf	I	2,87	4,38	**
				4_evfpj	I	2,87	4,38	**
				8_evfor	I	3,41	7,99	**
				1_ehpcfpj	III	2,87	184,38	**
				2_ehpcfpf	III	2,87	184,38	**
				6_evfpf	IV	3,67	348,97	**
			4_evfpj	3_evfbcr	I	2,87	85,62	**
				2_evfbc	III	2,76	268,34	**
				3_evfbcr	II	3,67	101,03	**
			8_evfor	3_evfbcr	I	3,41	82,01	**
		Horizontal Face Front Profile	1_ehpcfpj	2_evfbc	I	2,76	88,34	**
				3_evfbcr	III	2,87	265,62	**
			2_ehpcfpf	2_evfbc	I	2,76	88,34	**
				3_evfbcr	III	2,87	265,62	**
			3_ehpcfor	2_evfbc	I	2,76	88,34	**
				3_evfbcr	III	2,87	265,62	**
	Indication							
	Movement	Vertical-Front	1_mvfcp	2_mvptr	II	2,6	110,55	**
				5_mvfped	IV	2,82	320,72	**
			2_mvfbed	1_mvptbed	I	3,64	11,52	**
				2_mvptr	I	3,23	19,11	**
				4_mvfps	III	2,81	266,9	**
				5_mvfped	III	3,27	208,46	**
			4_mvfps	2_mvptr	II	2,65	120,38	**
				2_mvfbed	III	2,81	183,1	**
				5_mvfped	IV	2,83	329,85	**
				6_mvfpea	IV	3,71	342,14	**
				7_mvfmp	IV	2,98	337,55	**

			5_mvfped	7_mvfmp	I	2,65	37,4	**
				1_mvfcp	II	2,82	129,28	**
				4_mvfps	II	2,83	120,15	**
				2_mvfbed	III	3,27	241,54	**
			6_mvfpea	4_mvfps	II	3,71	107,86	**
			7_mvfmp	5_mvfped	I	2,65	52,6	**
				10_mvfppe	I	2,75	30,58	**
				4_mvfps	II	2,98	112,45	**
			10_mvfppe	7_mvfmp	I	2,75	59,42	**
			11_mvffba	1_mvptbed	I	3,11	77,17	**
		Complete Vertical Profile	1_mvptbed	2_mvfbed	I	3,64	78,48	**
				8_mvffpc	IV	2,76	350,94	**
				12_mvffba	IV	3,1	356,06	**
			2_mvptr	2_mvfbed	I	3,23	70,89	**
				1_mvfcp	IV	2,6	339,45	**
				4_mvfps	IV	2,65	329,62	**
Character Wolf	Macrocategory	Category	Focal behavior	Conditioned behavior	Significant vectors			
					Quadrant	Length	Angle	Significance
	Estatic							
	Indication	Horizontal Face Front Profile	1_ihptice	2-ihpcficecg	IV	3,01	293,48	**
	Movement	Vertical-front	1_mvfcp	3_mvfbea	I	2,6	114,35	**
				8_mvffpc	II	2,62	320,72	**
				1_mvptbed	II	2,65	114,35	**
				2_mvptr	II	2,65	114,35	**
				1_mhpcfppe	I	3,08	4,98	**
				2_mhpcfca	III	2,81	244,94	**
				1_mhptr	II	2,74	113,26	**
			3_mvfbea	1_mvfcp	I	2,6	87,83	**
				4_mvfps	II	4,21	106,94	**
				7_mvfmp	IV	2,88	338,14	**
				1_mhpcfppe	I	3,84	51,08	**
			4_mvfps	3_mvfbea	IV	4,21	343,06	**
				1_mhpcfppe	IV	2,64	328,89	**
			5_mvfped	2_mhpcfca	I	2,67	63,36	**
			7_mvfmp	3_mvfbea	II	2,88	11,86	**
				2_mhpcfca	IV	3,64	333,51	**
			8_mvffpc	1_mvfcp	IV	2,62	335,65	**
		Complete Vertical Profile	2_mvptr	1_mvfcp	IV	2,65	335,92	**
				8_mvffpc	IV	2,76	350,94	**
				12_mvffba	IV	3,1	356,06	**
		Horizontal Face Front Profile	1_mhpcfppe	1_mvfcp	I	3,08	85,02	**
				3_mvfbea	I	3,84	38;92	**
				4_mvfps	II	2,64	121,11	**

			2_mhpcfca	1_mvfcp	III	2,81	205,06	**
				5_mvfped	I	2,67	26,64	**
				7_mvfmp	II	3,64	116,49	**
		Complete Horizontal Profile	1_mhptr	1_mvfcp	IV	2,74	336,74	**
			2_mhptca	10_mvfr	IV	2,65	329,72	**
				2_mhpcfca	II	3,11	90,04	**
**6 AGE**								
Character Rabbit	Macrocategory	Category	Focal behavior	Conditioned behavior	Significant vectors			
					Quadrant	Length	Angle	Significance
	Estatic							
	Indication		ivficecg	Frente1_ivficecg	I	3,56	45	**
	Movement							
Character Wolf	Macrocategory	Category	Focal behavior	Conditioned behavior	Significant vectors			
					Quadrant	Length	Angle	Significance
	Estatic	Vertical-front	vfbc	2_vfbc	I	3,05	56,97	**
			vfpb	2_vfbc	I	2,98	90	**
	Indication							
	Movement	Vertical-front	mvfcp	2_mvptbea	I	2,98	65,56	**
			mvfbed	3_mhpcfpea	II	2,98	101,42	**
				5_mhpcffpc	II	2,98	101,42	**
			mvffbc	5_mhptfpc	I	4,24	90	**
				10_mhptca	II	2,87	94,75	**
			mvffpa	3_mhpcfpea	II	3,18	95,5	**
				5_mhpcffpc	II	3,18	95,5	**
		Vertical Face Front Profile	mvpcffba	5_mhptfpc	I	4,24	90	**
				10_mhptca	II	2,87	94,75	**
		Complete Vertical Profile	mvptbed	10_mhptca	IV	3,02	351,74	**
			mvptbea	1_mvfcp	I	2,98	24,44	**
		Horizontal Face Front Profile	mhpcfped	9_mhpcfoa	I	4,2	56,86	**
				3_mhptpea	II	3,54	98,05	**
			mhpcfpea	2_mvfbed	IV	2,98	348,58	**
				11_mvffpa	IV	3,18	354,5	**
			mhpcffpc	2_mvfbed	IV	2,98	348,58	**
				11_mvffpa	IV	3,18	354,5	**
			mhpcfoa	2_mhpcfped	I	4,2	33,14	**
				9_mhpcfoa	I	6,68	45	**
				3_mhptpea	I	4,82	75,63	**
				8_mhptr	IV	3,62	350,03	**
		Complete horinzontal Profile	mhptpea	2_mhpcfped	IV	3,54	351,95	**
				9_mhpcfoa	I	4,82	14,37	**
			mhptfpc	2_mvffbc	I	3,67	90	**
			mhptr	9_mhpcfoa	II	3,62	99,97	**
			mhptca	1_mvptbed	II	3,02	98,26	**

7 AGE								
Character Rabbit	Macrocategory	Category	Focal behavior	Conditioned behavior	Significant vectors			
					Quadrant	Length	Angle	Significance
	Estatic							
	Indication							
	Movement	Vertical-front	1_mvfcp	2_mvpcfps	II	2,64	97,58	**
				8_mhptoa	IV	2,64	352,42	**
			3_mvfbea	3_mvfbea	I	2,68	45	**
				2_mhpcffpc	I	3,06	24,01	**
			4_mvffba	6_mvfped	IV	3,03	342,98	**
			6_mvfped	4_mvffba	II	3,03	107,02	**
				6_mvfped	I	2,89	45	**
				2_mvptfbc	II	2,94	102,16	**
			8_mvfppe	2_mvptfbc	IV	3,31	342,76	**
		Vertical Face Front Profile	2_mvpcfps	1_mvfcp	IV	2,64	352,42	**
			4_mvpcffpc	6_mvfped	I	3,18	68,29	**
				6_mvpcffpa	IV	2,76	349,59	**
			6_mvpcffpa	6_mvfped	I	2,94	82,01	**
				4_mvpcffpc	II	2,76	100,41	**
			7_mvpcffbc	6_mvfped	I	4,32	76,19	**
		Complete Vertical Profile	2_mvptfbc	6_mvfped	IV	2,94	347,84	**
				8_mvfppe	II	3,31	107,24	**
		Horizontal Face Front Profile	1_mhpcfped	3_mhptpea	II	2,7	100,65	**
			2_mhpcffpc	3_mvfbea	I	3,06	65,99	**
				3_mhptpea	II	2,71	97,4	**
			3_mhpcfppe	3_mhptpea	II	2,7	100,65	**
		Complete horinzontal Profile	3_mhptpea	1_mhpcfped	IV	2,7	349,35	**
				2_mhpcffpc	IV	2,71	352,6	**
				3_mhpcfppe	IV	2,7	349,35	**
				8_mhptoa	II	2,64	97,58	**
			8_mhptoa	3_mhptpea	IV	2,64	352,42	**
Character Rabbit	Macrocategory	Category	Focal behavior	Conditioned behavior	Significant vectors			
					Quadrant	Length	Angle	Significance
	Estatic							
	Indication							
	Movement	Vertical Face Front Profile	2_mvptfbc	3_mhpcfppe	IV	2,67	345,12	**
				6_mhptca	I	2,82	85,97	**
			4_mvptped	3_mhpcfppe	IV	2,67	245,12	**
				6_mhptca	I	2,82	85,97	**
		Horizontal Face Front Profile	2_mhpcffpc	3_mhpcfppe	IV	2,71	345,34	**
			3_mhpcfppe	2_mvptfbc	II	2,67	104,88	**
				4_mvptped	II	2,67	104,88	**
				2_mhpcffpc	II	2,71	104,66	**

		Complete horinzontal Profile	4_mhptfpa	2_mvptfbc	IV	3,28	354,88	**
				4_mvptped	IV	3,28	354,88	**
			6_mhptca	2_mvptfbc	I	2,82	4,03	**
				4_mvptped	I	2,82	4,03	**
			7_mhptfba	2_mvptfbc	IV	3,28	354,88	**
				4_mvptped	IV	3,28	354,88	**
**8 AGE**								
Character Rabbitt	Macrocategory	Category	Focal behavior	Conditioned behavior	Significant vectors			
					Quadrant	Length	Angle	Significance
	Estatic							
	Indication							
	Movement	Vertical-front	3_mvfbea	4_mvffba	I	2,7	20,79	**
				8_mvptfpa	IV	3,23	319,15	**
			4_mvffba	3_mvfbea	I	3,39	72,88	**
				4_mvffba	IV	3,45	356,1	**
				5_mvpcfppe	IV	3,31	350,29	**
				8_mvptfpa	IV	3,2	336,66	**
			5_mvfps	2_mhpcfped	I	3,02	56,35	**
				4_mhpcfppe	I	3,02	56,35	**
			6_mvfped	2_mhpcfped	I	3,32	53,67	**
				4_mhpcfppe	I	3,32	53,67	**
			7_mvfpea	1_mhpcfps	II	2,79	121,2	**
		Vertical Face Front Profile	1_mvpcfppe	4_mvffba	II	3,1	101,55	**
		Complete Vertical Profile	2_mvptfbc	4_mhptfpa	IV	2,87	341,88	**
			7_mvptfba	6_mvpcfoa	I	2,71	25,33	**
				9_mvptpea	I	3,46	9,16	**
			8_mvptfpa	3_mvfbea	II	2,7	130,85	**
			9_mvptpea	7_mvptfba	I	3,46	80,84	**
		Horizontal Face Front Profile	1_mhpcfps	7_mvfpea	IV	2,79	328,8	**
			2_mhpcfped	5_mvfps	I	3,02	33,65	**
				6_mvfped	I	3,32	36,33	**
			4_mhpcfppe	6_mvfped	I	2,78	45,03	**
		Complete horinzontal Profile	4_mhptfpa	2_mvptfbc	II	2,82	104,6	**
Character Wolf	Macrocategory	Category	Focal behavior	Conditioned behavior	Significant vectors			
					Quadrant	Length	Angle	Significance
	Estatic							
	Indication							
	Movement	Vertical-front	1_mvfcp	5_mvfps	IV	3,19	349,45	**
				2_mvptfbc	IV	3,26	349,67	**
				6_mhpcfbc	IV	3,15	349,33	**
			4_mvffba	1_mhptps	I	3,81	61,08	**

				6_mhptca	I	3,12	70,93	**
			5_mvfps	1_mvfcp	II	3,19	100,55	**
				2_mvptfbc	II	2,89	96,56	**
				1_mhpcfps	IV	2,82	338,95	**
				2_mhpcfped	IV	3,05	342,19	**
				6_mhpcfbc	IV	2,92	353,51	**
			6_mvfped	7_mvptfba	II	2,66	104,67	**
				8_mvptfpa	II	2,73	110,9	**
				1_mhpcfps	IV	3,21	33,25	**
				2_mhpcfped	IV	2,67	329,96	**
			7_mvfpea	7_mvptfba	II	4,1	96,6	**
				8_mvptfpa	II	2,69	104,67	**
		Complete Vertical Profile	2_mvptfbc	1_mvfcp	II	3,26	100,33	**
				5_mvfps	IV	2,89	353,44	**
				6_mhpcfbc	IV	2,85	353,37	**
			5_mvptfpc	2_mhptped	IV	2,89	322,06	**
			6_mvptca	7_mhptfba	IV	2,62	345,08	**
			7_mvptfba	6_mvfped	IV	2,66	345,33	**
				7_mvfpea	IV	4,1	353,4	**
				1_mhptps	I	3,22	45,96	**
			8_mvptfpa	6_mvfped	IV	2,73	339,1	**
				7_mvfpea	IV	2,69	345,33	**
			9_mvptpea	4_mhpcfppe	I	3,19	8,78	**
		Horizontal Face Front Profile	1_mhpcfps	5_mvfps	II	2,82	111,05	**
				6_mvfped	II	3,21	116,75	**
			2_mhpcfped	5_mvfps	II	2,82	111,05	**
				6_mvfped	II	3,21	116,75	**
			4_mhpcfppe	9_mvptpea	I	3,19	81,22	**
				2_mhptped	IV	3	312,8	**
			6_mhpcfbc	1_mvfcp	II	3,15	100,67	**
				5_mvfps	II	2,92	96,49	**
				2_mvptfbc	II	2,85	96,63	**
		Complete horinzontal Profile	1_mhptps	4_mvffba	I	3,9	24,69	**
				7_mvptfba	I	3,22	44,04	**
				1_mhptps	I	6,07	45	**
				6_mhptca	I	4,91	46,4	**
			2_mhptped	5_mvptfpc	II	2,89	127,94	**
				4_mhpcfppe	II	3	137,2	**
			4_mhptfpa	4_mhptfpa	III	2,66	225	**
			6_mhptca	4_mvffba	I	3,24	14,97	**
				1_mhptps	I	4,91	43,6	**
				6_mhptca	I	2,76	45	**
			7_mhptfba	6_mvptca	II	2,62	104,92	**
			7_mhptfbc	1_mhptps	III	1	0,62	**
				6_mhptca	III	3,34	232,1	**

Figure S1. Examples of drawings made by children in the three macrocategories of study: (a) static; (b) indication; (c) movement.

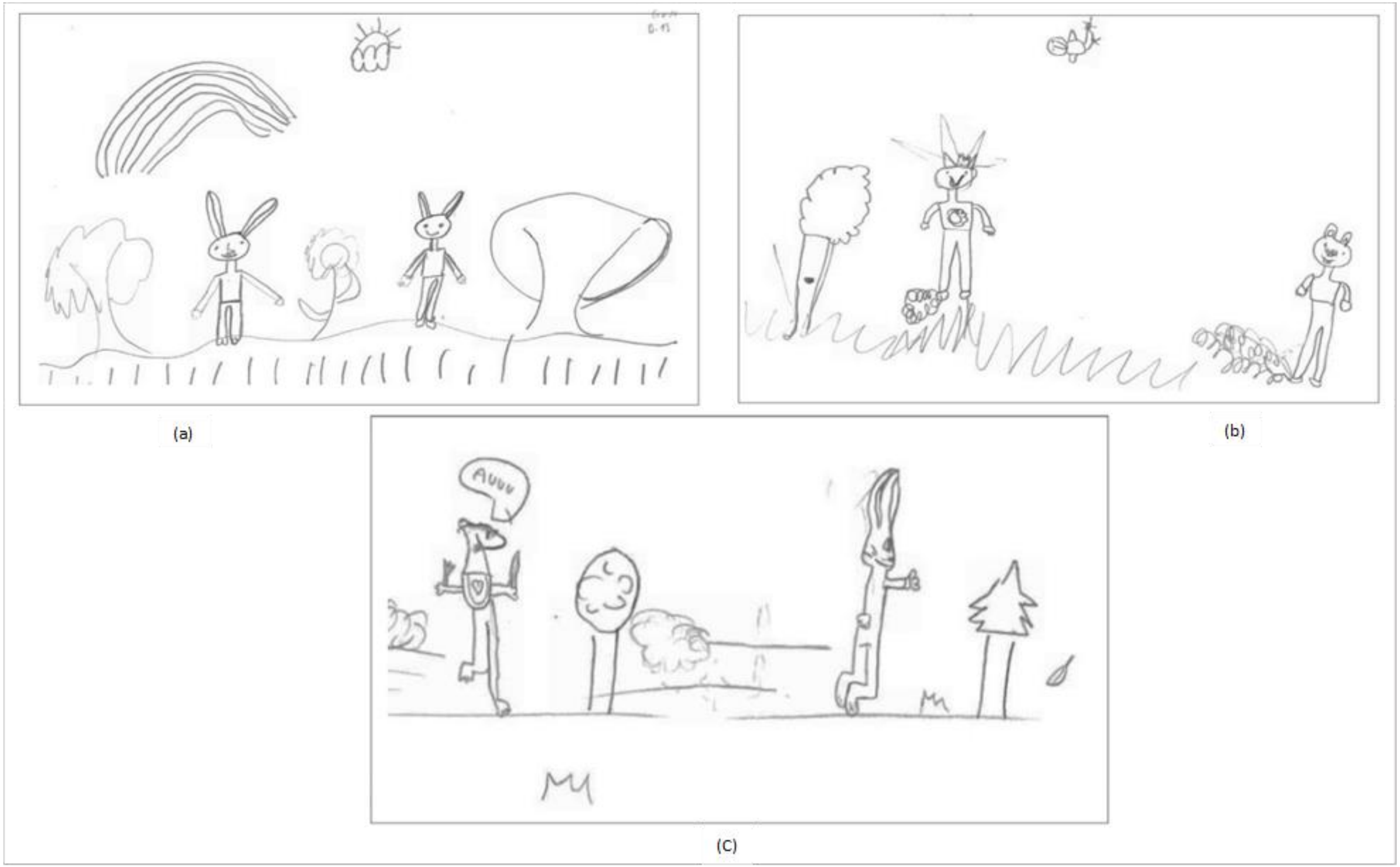

Supplement: Supplementary file 1 [file ijerph-19-02844-s001.zip › ijerph-1521618-Supplementary Materials.pdf]
